# Preoperative short-course radiotherapy in rectal cancer patients: results and prognostic factors

**DOI:** 10.1007/s13566-017-0340-5

**Published:** 2017-12-20

**Authors:** Tomasz Skóra, Jadwiga Nowak-Sadzikowska, Dariusz Martynów, Mariusz Wszołek, Beata Sas-Korczyńska

**Affiliations:** Krakow Branch, Department of Oncology, Maria Sklodowska-Curie Memorial Cancer Centre and Institute of Oncology, ul. Garncarska 11, 31-115 Kraków, Poland

**Keywords:** Rectal cancer, Short-course radiotherapy, Adjuvant radiotherapy, Combined modality treatment

## Abstract

**Objective:**

The purpose of this study was to evaluate the clinical outcome of preoperative short-course radiotherapy for rectal cancer patients.

**Methods:**

The study group comprised 210 patients with pathologically proven resectable rectal cancer. Between 2001 and 2013, they were treated preoperatively with short-course radiotherapy (25 Gy delivered in five fractions), followed by total mesorectal excision. Adjuvant 5-fluorouracil-based chemotherapy was administered at the discretion of the treating physician, depending on the pathological stage.

**Results:**

After a median follow-up of 57 months, the following 5-year survival rates were observed: overall survival—66.4%, disease-free survival—67.2%, locoregional relapse-free survival—91.7%, and distant metastases-free survival—71.5%. The local failure was observed in 15 patients. Ten patients (4.8%) achieved pathologic complete response. The multivariate analysis demonstrated the regional lymph node involvement to be statistically significant for unfavorable outcomes in terms of all estimated survival rates. Lymphovascular invasion was found to be a strong predictor of survival (HR = 1.68; 95% CI 1.29–3.55) and treatment failure (HR = 1.54; 95% CI 1.08–3.34). The presence of positive surgical circumferential margin was related to six times higher risk of locoregional recurrence. Early and late severe treatment-induced toxicity was reported in 1 and 7.6% patients, respectively.

**Conclusions:**

Preoperative short-course radiotherapy followed by total mesorectal excision and adjuvant chemotherapy allows to achieve excellent local control and favorable survival rates. The treatment-induced toxicity is acceptable.

## Introduction

Over the last few decades, parallel to irrefutable benefits from dynamic socioeconomic changes in Poland, one can observe undesirable effects typical for highly developed countries. One of the most serious is rapidly rising incidence of lifestyle diseases, among which a significant percentage is represented by oncological diseases.

In 2014, cancer accounted for about 25% of all deaths recorded in Poland. Approximately 12% of these deaths were caused by the lower gastrointestinal neoplasms, including rectum cancer. Colorectal cancer is one of the most commonly diagnosed diseases in both sexes, classified in the 2nd and 3rd positions in women and men, respectively. A similar situation is reported in mortality rates. In the case of Poland only half a century was needed to switch from the lowest rate in Europe to the values observed in western countries [1, 2].

In 2013, according to the National Cancer Registry, nearly 5900 new rectal cancer cases were diagnosed and more than 3300 deaths resulting from rectal cancer were noted [2].

The abovementioned epidemiological data indicate a crucial role for the most efficient treatment determination in this large group of patients. At the same time, there is still a lot of controversies relating to the optimal treatment course. For years, total mesorectal excision (TME) has been the gold standard for the surgical management of rectal cancer. Introduction of this procedure to surgical practice has resulted in significant improvements in local cure rates, compared to conventional surgical techniques. Nevertheless, local recurrences continue to be a serious clinical problem, due to their significant symptoms and low efficiency of secondary therapeutic options. For this reason, adjuvant management remains an important component of treatment for patients with rectal cancer.

The results of randomized trials, on patients with resectable rectal cancer, have unequivocally confirmed the benefit of adjuvant radiotherapy [3–6]. Depending on the clinical situation, two radiotherapy strategies are currently in use, preoperative and postoperative, both concurrently combined with chemotherapy.

Preoperative radiotherapy, which is the preferred one, is associated with significant improvement in local control rates and better early and late treatment tolerance compared to postoperative radiotherapy [7, 8]. Based on experience, many European centers prefer the so-called short preoperative irradiation. This is due to its favorable toxicity profile and comparable efficacy to “long” conventionally fractionated radiochemotherapy [9–12].

The objective of this study is to evaluate long-term results of short preoperative radiotherapy regimen in patients with rectal cancer treated in our center.

## Material and methods

Between 2001 and 2013, 424 rectal cancer patients were treated with short preoperative radiotherapy at the Maria Sklodowska-Curie Memorial Institute of Oncology in Kraków. Further analysis was focused on a group of 210 (49.5%) patients, whose entire therapeutic process (radiotherapy, surgery, and chemotherapy) was performed at our center.

Eligible patients were those with (1) histopathologically proven rectal adenocarcinoma; (2) clinical T3 or/and cN(+) stage; (3) clinical T4 stage with contraindications for concomitant radiochemotherapy; (4) resectable tumor located within 15 cm from the anal verge as measured by flexible rectoscopy; and (5) no evidence of distant metastases.

All patients underwent preoperative radiotherapy using a short-course irradiation regimen with a total dose of 25 Gy given in five fractions over 5 days. The clinical target volume included the primary tumor, the mesorectal and presacral lymph nodes, the lymph nodes along the internal iliac vessels up to the promontory level, and the lymph nodes at the obturator foramen. The treatment was delivered with the three- or four-beam technique with the patient lying either supine or prone.

In 27 cases, the total treatment time was longer due to a holiday or therapeutic break, yet not exceeding a period of 8 days. Three patients did not receive a full course of radiotherapy for technical (one patient—15 Gy in three fractions) or medical reasons (two patients—20 Gy in four fractions). No concomitant chemotherapy was given.

All patients underwent surgical treatment. The most common types of surgery were lower anterior resection (61.9%) and abdominoperineal resection (29%); in 14 (6.7%) patients, Hartman’s procedure was carried out. In two cT1N0 cases (1%), local tumor excision was performed after radiotherapy. In another patient, because of large intestine polyposis, total proctocolectomy was performed. Due to the locally advanced and unresectable tumor, two patients underwent palliative surgery with colostomy formation.

The median time between the end of radiotherapy and the operation was 13 days. In 71 (33.8%) patients, surgery was performed within 10 days after radiotherapy; for the remaining 139 patients (66.2%), the break between radiotherapy and surgery was above 10 days.

A histopathological evaluation of the postoperative material revealed adenocarcinoma in 200 (95.2%) cases. In the remaining 10 (4.8%) patients, a complete regression of cancer was confirmed.

Table 1 summarizes the characteristics of the patient, tumor, and surgical treatment.

**Table 1:** Presurgical patient, tumor, and treatment characteristics

Characteristic	Number	Percentage
Age		
Median (years)	64	
Range (years)	30–85	
Gender		
Female	87	42.2
Male	123	57.8
Karnofsky Performance Score		
< 80%	45	21.4
≥ 80%	165	78.6
Tumor distance from anal verge		
≤ 5 cm	83	39.5
6–10 cm	110	52.4
> 10 cm	17	8.1
Hemoglobin		
< 12 g/dl	49	23.3
≥ 12 g/dl	134	63.8
Unknown	27	12.9
Type of rectal surgery		
Anterior resection	130	61.9
Abdominoperineal resection	61	29.0
Other	19	9.1
pT stage		
pT0	10	4.8
pT1	9	4.3
pT2	48	22.8
pT3	133	63.3
pT4	8	3.8
pTx	2	1.0
pN stage		
pN0	120	57.1
pN1	56	26.7
pN2	26	12.4
pNx	8	3.8
Circumferential resection margins status		
Close/positive	17	8.1
Negative	191	90.9
Extracapsular extension		
Yes	16	7.6
No	186	88.6
Lymphovascular invasion		
Yes	18	8.6
No	190	90.4
Tumor grade		
G1	33	15.7
G2	110	52.4
G3	11	5.2
G4	1	0.5
Gx	55	26.2

In 69 (32.9%) patients, adjuvant systemic treatment, most often 5-fluorouracil and leucovorin (65 patients) regimen, was administered. Fifty-six (26.7%) patients received the full regimen of six chemotherapy cycles.

The results were evaluated in terms of overall survival (OS), disease-free survival (DFS), locoregional relapse-free survival (LRRFS), and distant metastasis-free survival (DMFS). The 5-year survival rates were estimated using the Kaplan-Meier method.

The influence of selected factors on the patient prognosis was assessed with the Cox proportional hazards model. The effectiveness of radiotherapy was evaluated, depending on the parameters characterizing patient (age, sex, performance status by Karnofsky Performance Score, pretreatment hemoglobin level), tumor (pTNM, differentiation grade, lymphovascular invasion, extracapsular extension, distance from the anal verge, pathologic complete response to preoperative radiotherapy—pCR), and treatment characteristics (type of surgery, surgical margin status).

Statistical analyses were performed using STATISTICA, version 12 (StatSoft, Inc., 2014).

Acute and late treatment toxicity was assessed according to CTCAE version 4.0.

## Results

Median follow-up calculated from the date of surgery was 57 months (range 1 to 178 months).

The locoregional tumor recurrence was found in 17 (8.1%) patients. It has occurred between 5 and 89 months after surgery (median time 21 months) and most often had a local character (13 patients). In the remaining four patients, there were two locoregional failures and two other patients had nodal recurrence. In 17 (8.1%) cases during primary surgery, no radical microscopic circumferential resection margin was achieved.

Distant dissemination was reported in 55 (26.2%) patients. In 52, that was the first symptom of failure. Most often, metastatic lesions were located in the liver, lung, and distant lymph nodes.

**Fig. 1:**
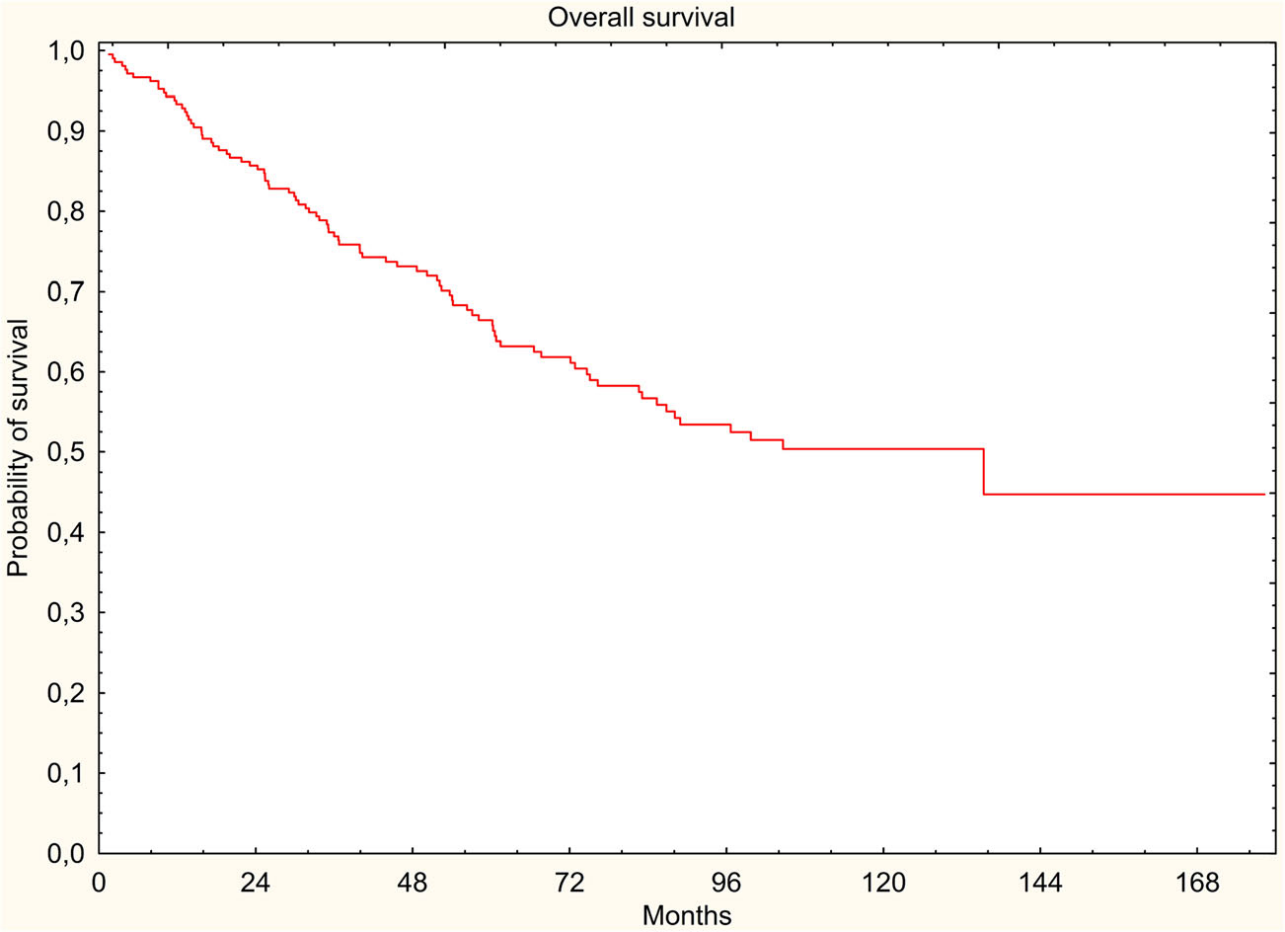
Overall survival (Kaplan-Meier curve)

**Fig. 2:**
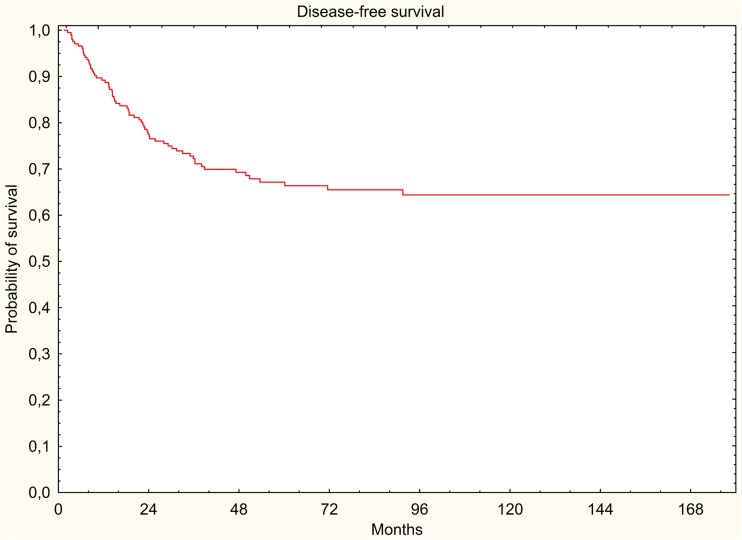
Disease-free survival (Kaplan-Meier curve)

**Fig. 3:**
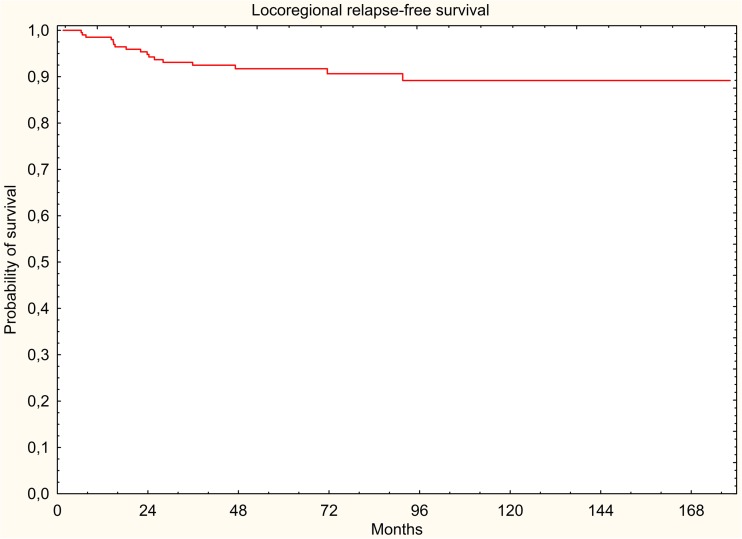
Locoregional relapse-free survival (Kaplan-Meier curve)

**Fig. 4:**
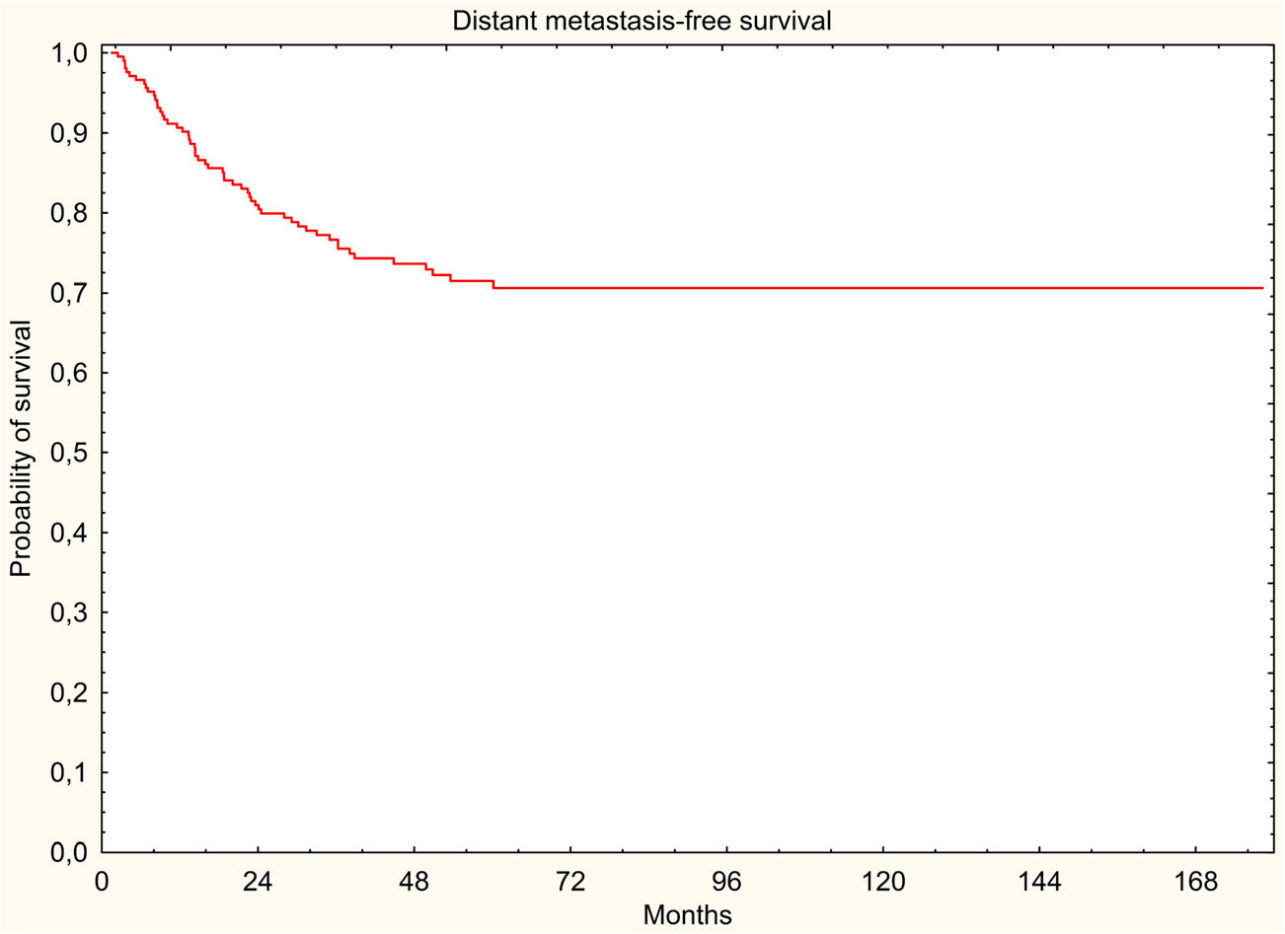
Distant metastasis-free survival (Kaplan-Meier curve)

In the study group, 88 (41.9%) deaths were documented, of which 58 (27.6%) were cancer-related deaths. All survival rates were measured from the start of radiotherapy. Five-year DFS, LRRFS, and DMFS rates were 67.2, 91.7, and 71.5%, respectively. Five-year OS rate was 66.4% (Figs. 1, 2, 3, and 4).

Multivariate analysis confirmed the prognostic significance of regional lymph node involvement for all of the assessed survival rates. Both OS and DFS were negatively influenced by the presence of lymphovascular invasion, with the HR of 1.68 (95% CI 1.29–3.55) and 1.54 (95% CI 1.08–3.34), respectively. Circumferential margin involvement was the strongest predictor of locoregional recurrence. The risk of locoregional failure was more than six times higher than in the group of tumor-free surgical margin patients. The risk of distant spread was the highest in the case of locally advanced tumors (HR = 4.37; 95% CI 1.59–11.98) and poorly differentiated rectal cancer (HR = 2.67; 95% CI 1.14–6.27). Detailed results are presented in Table 2.

**Table 2:** Multivariate Cox regression analysis

Survival	Variables	HR	CI 95%	*p* value
OS	age > 64 years	2.33	1.45–3.74	0.0004
	pN+	2.02	1.29–3.2	0.0023
	LVSI(+)	1.68	1.29–3.55	0.0008
	KPS ≤ 70%	1.45	1.13–4.95	0.0181
DFS	pT3–4	3.59	1.54–8.4	0.0031
	pN(+)	2.57	1.46–4.5	0.0011
	Mucinous component	2.25	1.31–3.86	0.0034
	LVSI(+)	1.54	1.08–3.34	0.0185
LRRFS	CRM(+)	6.45	2.37–17.6	0.0003
	pN(+)	3.5	1.2–10.0	0.0199
DMFS	pT3–4	4.37	1.59–11.98	0.0042
	G3–4	2.67	1.14–6.27	0.0237
	pN(+)	2.55	1.39–4.66	0.0024
	Mucinous component	2.54	1.44–4.49	0.0014

*OS* overall survival, *DFS* disease-free survival, *LRRFS* locoregional relapse-free survival, *DMFS* distant metastases-free survival, *LVSI* lymphovascular invasion, *KPS* Karnofsky Performance Score, *CRM* circumferential resection margin

Early radiation-induced toxicity of gastrointestinal and urinary tracts were observed in 20 (9.5%) patients, mostly of mild and moderate intensity. Severe grade 4 toxicity occurred in two (1%) patients, as increased mucous rectal discharge with bleeding and generalized peritonitis. Late treatment-induced toxicity was reported in 51 patients (24.3%), including 16 (7.6%) patients developing grade 3 toxicity (Table 3).

**Table 3:** Radiation-related G3/G4 toxicity (CTCAE v.4.0)

	Number
Acute toxicity	
Ileus/peritonitis	1
Gastrointestinal hemorrhage	1
Late toxicity	
Rectal pain	4
Anal stenosis	1
Diarrhea	1
Rectal tenesmus	1
Fecal incontinence	2
Intestinal stenosis	2
Rectovesical fistula	1
Pollakiuria	1
Urinary incontinence	2
Lower back pain	1

## Discussion

Stage II and III of rectal cancer are recognized in nearly 75% of patients with metastases-free rectal cancer [13]. In order to ensure the optimal therapeutic effect in this group of patients, it is necessary to implement multicomponent therapy. Over the last decades, opinions about the type of treatment and sequence of its individual components have been constantly evolving.

Based on the available data gathered from several randomized controlled trials, preoperative radiotherapy alone (25 Gy in 5 fractions) or in combination with chemotherapy (45–50.4 Gy in 25–28 fractions) is the widely accepted gold standard treatment. Compared to adjuvant therapy, it significantly reduces the percentage of local failures, without showing differences in overall survival. Sauer et al. reported a 5-year reduction in local recurrence rates by more than 50% (6 vs. 13%, *p* = 0.006) in patients treated with preoperative chemoradiotherapy compared with the same treatment administered postoperatively [7]. The observed benefit in terms of local control has been confirmed in long-term follow-up, where after 11 years the rate of local recurrence was 7.1 and 10.1%, respectively (*p* = 0.048) [8]. Additionally, in the case of neoadjuvant therapy, the authors observed early and late grade 3 and 4 radiation-induced toxicity less frequently [7]. Similar conclusions have been drawn by Sebag-Montefiore who compared a short preoperative regimen (25 Gy in five fractions) with adjuvant chemoradiotherapy. The local failure rates were 4.4 and 10.6%, respectively [14].

In the discussed series, local recurrence rate was 7.1% and is comparable with the one described in literature. All these failures were observed before 2009, when a large-scale preoperative diagnostics, including magnetic resonance imaging, was incorporated. This allowed for a significant reduction in the risk of locoregional stage underestimation and thus the more appropriate patient qualification to the correct therapeutic regimen.

The efficacy of preoperative radiotherapy in high-risk patients has been unequivocally confirmed in the Swedish Rectal Cancer Trial. The authors demonstrated statistically significant reduction in local failure rate (11 vs. 27%, *p* < 0.001) and an improvement in overall 5-year survival (58 vs. 48%; *p* = 0.004) compared to patients treated with surgery alone. This advantage was confirmed in the further course of observation. Overall survival rate after 13 years was 38 and 30%, respectively (*p* = 0.008). However, it should be stressed that the TME procedure was not used in the study [4, 5].

In the described group, the 5-year disease-free survival and overall survival rates were 67.2 and 66.4%, respectively. It is comparable to the results presented in the literature [3, 5, 12, 15, 16].

Since the obvious superiority of TME over the hitherto used surgical techniques has been demonstrated, the importance of adjuvant radiotherapy has slightly decreased. None of the conducted studies have confirmed the statistically significant improvement in overall survival. At the same time, high efficacy of preoperative radiotherapy was observed in prevention of local recurrence. In the Danish study, the use of short-course radiotherapy before TME was associated with a twofold reduction in local recurrence risk (12 vs. 6%) 5 years after the end of the treatment [3]. This benefit was still apparent 10 years later (11 vs. 5%; *p* < 0.0001) [17].

Results of the previous studies evaluating the effectiveness of preoperative therapy in resectable rectal cancer patients show no clear advantage of any of the neoadjuvant radiotherapy regimens. In addition to tumor downsizing and increased rate of complete response, there were no statistically significant differences in overall survival, local or distant recurrence rates, sphincter preservation rate, and late complications observed [12, 15].

It should be emphasized that the difference in pCR rates and consequently the potentially higher rate of sphincter-preserving surgery result not from the fractionation method of ionizing radiation, but from the time interval between radiotherapy and surgical treatment.

Complete pathological response is rarely observed (0–1.7%) in the group of patients operated shortly after radiotherapy completion [12, 18–21]. With a sufficiently long time of 4–6 weeks, also a short preoperative irradiation regimen provides an opportunity to increase the incidence of complete tumor regression rate (about 15%) [21, 22]. This has been confirmed in our study, where we observed 10 patients with complete tumor regression in postoperative material. In all cases, this time, ranging from 18 to 53 days, was significantly longer than the commonly recommended 7–10 days.

The problem of the optimal interval between radiotherapy and surgery has been partially solved by the findings of the Stockholm III Trial. The study aimed to compare three different schedules of preoperative radiotherapy (short-course radiotherapy, short-course radiotherapy with delay, and long-course radiotherapy with delay). The results confirmed a similar oncological efficacy of all analyzed radiotherapy schedules [16]. Time interval extension from 1 to 4–8 weeks allowed for a nearly sevenfold increase in the pCR rate in the group of patients treated with short-course radiotherapy (1.7 vs. 11.8%; *p* = 0.001) [19].

The treatment tolerance in the discussed group was good. Radiation-induced toxicity was predominantly mild and moderate. The incidence of severe complications in our study, both early (1%) and late (7.6%), did not differ from the literature [12, 16, 23, 24].

The impressive development of diagnostic and surgical techniques, resulting in treatment outcome improvement and reduction in perioperative complications, has partially reduced the role of neoadjuvant radiotherapy in the treatment of rectal cancer. At the same time, increased interest in minimally invasive surgical techniques (TEM, laparoscopy, local excision of the tumor) is noted. Undoubted advantage of these methods is marked improvement in their safety profile compared to traditional surgery. On the other hand, limiting the range of the operating field may result in an increased rate of locoregional recurrence [25–28]. This again gives new possible reasons for implementation of preoperative radiotherapy in order to minimize the risk of therapy failure.

## Conclusion

Taking into account the rapid increase in morbidity and death rate, rectal cancer is undoubtedly one of the biggest challenges for oncological care in developing countries such as Poland. In the face of unfavorable epidemiological prognosis, instant response to scientific reports appearing on a regular basis is indispensable, in order to effectively modify the therapeutic regimen and to ensure optimal treatment outcomes for this group of patients.

At the moment, a short-course preoperative radiotherapy is an important component of rectal cancer management. It improves the results of treatment in patients with locally advanced rectal cancer while providing a favorable and fully acceptable toxicity profile. An additional advantage is the short hospitalization time, making it a convenient and economically beneficial therapeutic option.
